# Trade‐offs and mixed infections in an obligate‐killing insect pathogen

**DOI:** 10.1111/1365-2656.12547

**Published:** 2016-06-13

**Authors:** Elizabeth M. Redman, Kenneth Wilson, Jenny S. Cory

**Affiliations:** ^1^ Molecular Ecology and Biocontrol GroupNERC Centre for Ecology and HydrologyMansfield RoadOxfordOX1 3SRUK; ^2^ Lancaster Environment CentreLancaster UniversityLancasterLA1 4YQUK; ^3^ Department of Biological SciencesSimon Fraser University8888 University DriveBurnabyV5A 1S6BCCanada; ^4^ Present address: Faculty of Veterinary MedicineUniversity of Calgary3330 Hospital Drive NWCalgaryABT2N 4N1Canada

**Keywords:** dose–response, entomopathogen, infection diversity, mortality rate, polymorphism, transmission potential, virulence

## Abstract

1. Natural populations of pathogens are frequently composed of numerous interacting strains. Understanding what maintains this diversity remains a key focus of research in disease ecology. In addition, within‐host pathogen dynamics can have a strong impact on both infection outcome and the evolution of pathogen virulence, and thus, understanding the impact of pathogen diversity is important for disease management.
2. We compared eight genetically distinguishable variants from *Spodoptera exempta* nucleopolyhedrovirus (SpexNPV) isolated from the African armyworm, *Spodoptera exempta*. NPVs are obligate killers, and the vast majority of transmission stages are not released until after the host has died.
3. The NPV variants differed significantly in their virulence and could be clustered into two groups based on their dose–response curves. They also differed in their speed of kill and productivity (transmission potential) for *S. exempta*. The mixed‐genotype wild‐type (WT) SpexNPV, from which each variant was isolated, was significantly more virulent than any individual variant and its mean mortality rate was within the fastest group of individual variants. However, the WT virus produced fewer new infectious stages than any single variant, which might reflect competition among the variants.
4. A survival analysis, combining the mortality and speed of kill data, confirmed the superiority of the genetically mixed WT virus over any single variant. *Spodoptera exempta* larvae infected with WT SpexNPV were predicted to die 2·7 and 1·9 times faster than insects infected with isolates from either of the two clusters of genotypes.
5. Theory suggests that there are likely to be trade‐offs between pathogen fitness traits. Across all larvae, there was a negative linear relationship between virus yield and speed of kill, such that more rapid host death carried the cost of producing fewer transmission stages. We also found a near‐significant relationship for the same trend at the intervariant level. However, there was no evidence for a significant relationship between the induced level of mortality and transmission potential (virus yield) or speed of kill.

## Introduction

Pathogens (micro‐parasites) are ubiquitous in animal and plant populations and often cause acute infections that can result in high levels of mortality and devastating epizootics (e.g. Jones *et al*. 2008). Pathogen populations are often composed of multiple strains (Read & Taylor 2001) and molecular and genomic analyses are showing that mixed‐genotype, as well as mixed‐species, infections are common (Balmer & Tanner 2011; Liu, Chen & Bonning 2015). Genetic diversity in pathogens may be generated by mutation or, more frequently, recombination, over relatively short periods of time, even during the course of infection within a single host. However, we still know little about temporal and spatial patterns of pathogen diversity in natural populations and the mechanisms that act to maintain it (Hodgson *et al*. 2001; Lively *et al*. 2014). In addition, multiple infections are predicted to have a major impact on the evolution of virulence and pathogen transmission (Alizon, de Roode & Michalakis 2013). Therefore, understanding the nature and consequences of mixed infections is important for disease mitigation and potential virulence management (Read & Taylor 2001; Andre & Hochberg 2005).

Pathogen (and host) diversity could be maintained by a variety of processes, including genotype × genotype interactions and negative frequency‐dependent selection, trade‐offs between virulence traits or differential selection (Hodgson *et al*. 2003; Lively *et al*. 2014). For pathogens, there are additional mechanisms for promoting diversity that could come about through beneficial interactions between genotypes during co‐infection, and the presence of deletion mutants or defective interfering particles, which may act as cheating genotypes (Hodgson *et al*. 2003). Some of these evolutionary mechanisms have been supported through studies on a limited number of host–parasite systems (e.g. Koskella & Lively 2009; Wolinska & King 2009; Clavijo *et al*. 2010). However, how they apply to a broader range of species, their relative importance and the conditions under which they are most prevalent are still not clear (Lively *et al*. 2014).

Insects are infected by a wide range of pathogens, some of which can produce impressive epizootics (Cory & Myers 2003; Hajek 2004). With asexual microparasites, it might be expected that the fittest genotype would dominate an infection within a single host or an epizootic (Hodgson *et al*. 2001); however, entomopathogen heterogeneity is often very high. What mechanisms drive this high level of genotypic and phenotypic diversity? Many of the better‐studied insect pathogens are obligate killers. Obligate killers must be highly virulent and kill the host to be horizontally transmitted; otherwise, the cost is severe. Why particular pathogens evolve to a particular level of virulence (defined here as the degree of harm to their hosts, Alizon *et al*. 2009; but see discussion in Thomas & Elkinton 2004; Shapiro‐Ilan *et al*. 2005) is one of the most pervasive questions in evolutionary biology and has generated a rich theoretical literature (e.g. Ebert & Weisser 1997; Alizon *et al*. 2009). The dominant (but not the only) model for the evolution of virulence assumes a trade‐off between pathogen traits, in particular, virulence and transmission, such that more rapid host exploitation, which results in higher transmission, comes at the cost of increasing host mortality, which would curtail the transmission process (Frank 1996; Alizon *et al*. 2009). Virulence should thus evolve to a maximum or an intermediate level depending on the shape of the virulence–transmission relationship. Obligate killers represent an interesting test of the trade‐off hypothesis as the death of the host promotes, rather than curtails, transmission, and it is not clear whether there is a cost of virulence (Ebert & Weisser 1997), and whether the link between rate of host exploitation and virulence might be uncoupled. However, evidence supporting the trade‐off hypothesis has been found in two obligate‐killing, invertebrate pathogens (a bacterium in the crustacean, *Daphnia magna*, Jensen *et al*. 2006; and a microsporidian fungus in the beetle, *Tribolium castaneum*, Bérénos, Schmid‐Hempel & Wegner 2009). Further studies are needed to test the generality of these results and to establish whether trade‐offs in fitness parameters might contribute to the maintenance of variation in invertebrate pathogen populations.

A particularly diverse group of obligate killers are the baculoviruses, a family of DNA viruses which have only been isolated from insects, particularly Lepidoptera (Cory & Myers 2003). Baculoviruses show high levels of genetic variation (e.g. Laitinen, Otvos & Levin 1996; Cooper, Cory & Myers 2003), and this variation translates into differences in phenotype (Hodgson *et al*. 2001; Cory *et al*. 2005; Murillo *et al*. 2006). Available evidence indicates that diversity and population structure are important in baculoviruses: a mixed infection of two baculovirus genotypes produced higher levels of mortality in a lepidopteran host than either genotype alone (Hodgson *et al*. 2004). Baculovirus populations can also include genotypes that contain deletions in their genome that can only be transmitted with a complete virus (Muñoz, Castillejo & Caballero 1998; Simón *et al*. 2006). This is possible because the most common type of baculovirus, a nucleopolyhedrovirus (NPV), has a unique morphology whereby many virus particles (which can themselves contain multiple genomes) are embedded in a proteinaceous matrix (the occlusion body, OB). The OB is the transmission stage of the baculovirus and needs to be ingested by a susceptible larva for infection to occur. Thus, each OB could contain multiple genotypes; this is thought to be an adaptive mechanism for maintaining diversity during horizontal transmission (Clavijo *et al*. 2010). However, few studies have been directed at exploring this variation in natural populations, the mechanisms that maintain this variation, or its relevance for infection dynamics and the evolution of virulence.

In the majority of Lepidoptera, baculovirus infections are highly productive and infect most body tissues, usually resulting in larval disintegration and the release of millions of transmission stages, which are then available to be ingested by other susceptible larvae. Horizontal transmission is the main route of transmission during epizootics. Vertical transmission does occur; however, this is usually at very low levels and appears to vary considerably between host–virus combinations (Kukan 1999; Burden *et al*. 2002). A faster speed of kill (shorter duration of infection) could potentially result in more rounds of infection in new, susceptible hosts. However, there are costs to this. Speed of kill clearly affects the production of transmission stages by altering the amount of tissue available for conversion into OBs; the longer the insect grows and remains a larva, the more the OBs will be produced. A baculovirus is therefore likely to maximize OB production up until a level where host growth rate and conversion of tissue to virus no longer increases unless there are costs to doing this (see also Ebert & Weisser 1997). As baculoviruses only infect the larval stages of Lepidoptera, the cut‐off point would therefore be metamorphosis into a pupa. However, optimum speed of kill is harder to predict. Ebert & Weisser (1997) examined the impact of parasite‐independent mortality on optimal killing time. In addition, speed of kill (and the release of transmission stages) is likely to depend on host ecology and the opportunities for transmission to new hosts, as the viability of many microparasite transmission stages is severely reduced by ultraviolet irradiation. For individual variants, evidence to date indicates that OB production increases linearly with increasing time to death (e.g. Hernández‐Crespo *et al*. 2001; Georgievska *et al*. 2010) although there is some evidence that this plateaus at longer speeds of kill (Hodgson *et al*. 2001). Evidence for trade‐offs in any of these traits among baculovirus variants has not previously been demonstrated (Hodgson *et al*. 2004).

Our study system is the African armyworm, *Spodoptera exempta* (Walk.) and its NPV (SpexNPV). *Spodoptera exempta* is found predominantly on the eastern side of the African continent, where it is a major pest of various graminaceous crops such as maize, sorghum, millet and also pasture grasses (Rose, Dewhurst & Page 2000). *Spodoptera exempta* larvae exhibit two distinct phases, a solitary form which occurs at low densities especially during the dry season, and a darker, gregarious morph which can occur at very high population densities (hence the armyworm epithet) causing irregular pest outbreaks during the rainy season. This species is highly migratory and can travel thousands of kilometres during a field season, while undergoing numerous generations, and crop damage can be extensive. NPV is frequently associated with high‐density populations, particularly later in the season (Brown & Swaine 1965; Rose, Dewhurst & Page 2000; Graham *et al*. 2012). Recent studies on *S. exempta* populations in Tanzania have demonstrated that vertical transmission (transfer from parent to offspring) of covert NPV is very high, approaching 100% (Vilaplana *et al*. 2010) and that the two phases differ in their susceptibility to NPV and their capacity to transmit the virus vertically as an overt infection (Reeson *et al*. 2000; Vilaplana *et al*. 2008). Genetic variation of NPV in this system is very high (Redman *et al*. 2010); however, the presence of genetic variation alone does not necessarily translate into differences in the phenotype of the virus variants. Based on earlier studies, we would predict that this genetic variation would translate into differences in virulence determinants: one of the assumptions underlying the trade‐off theory. Investigation of multiple traits in numerous variants allows us to examine potential trade‐offs and whether they are influenced by inoculum size. As baculoviruses infect only the larval stage, pathogen growth will not be competing directly with host reproduction, and thus, the pathogen could maximize its productivity. We therefore hypothesized that there were unlikely to be trade‐offs between the level of fatal infection and either the speed of kill or production of transmission stages. Finally, as genetic variation is frequently very high in baculoviruses, we predicted that there would be benefits to mixed‐genotype infections. To test these ideas, we estimated three parameters which contribute to virus fitness: host mortality and speed of kill were measured as estimates of virus virulence, and the number of OBs yielded at host death was estimated as a correlate for virus transmission potential, as host mortality is dose dependent (Reeson *et al*. 1998). Our aims were to (i) quantify the extent of genetic variation in key NPV fitness traits; (ii) to determine whether there are trade‐offs between the different fitness measures; and (iii) to establish whether the performance of the (parental) mixed‐genotype, wild‐type (WT) virus is an additive function of the fitness traits of the individual variants it comprises or whether there is any evidence for cooperation or interference between the variants.

## Materials and methods

### Host–Pathogen System

The original sample of *S. exempta* NPV (SpexNPV) WT virus was collected from *S. exempta* populations in Tanzania in 1972. A preliminary study of this isolate, using restriction fragment profiling of the DNA, revealed numerous submolar bands, indicating the presence of multiple ‘variants’ (genotypes). The variants were separated by *in vivo* cloning in *S. exempta*. *In vivo* cloning involves challenging individual larvae with a pathogen dose that will result in low levels of mortality (ideally 10% or less), under the assumption that infections at this level will result from a single virus OB (see Zwart *et al*. 2009). Each variant used in the study went through two rounds of low‐dose *in vivo* cloning plus two rounds of amplification. Variant stability at each stage was confirmed by restriction fragment profiling of viral DNA (see Redman *et al*. 2010 for details). Eight genotypic variants were selected for comparison, labelled as A, B, C, D, E, F, G and H [these are the same as SpexNPV variants 1, 3, 6, 7, 11, 12, 14 and 16, respectively, in Redman *et al*. (2010)]. We chose *in vivo* cloning, rather than *in vitro* cloning, because we did not want to select variants that were adapted to cell culture. In addition, there is good evidence that the passage of baculoviruses in cell culture can generate variants with sizeable deletions (Piljman *et al*. 2001). However, as NPVs contain multiple virions within an OB, and each virion can potentially contain several nucleocapsids, *in vivo* cloning cannot guarantee that each variant is a true clone (as compared to plaque‐picked variants grown *in vitro*), and it is possible that more than a single clone could be present within each variant. However, if present, these would be at very low levels and are unlikely to produce biological effects over one round of infection.

The *S. exempta* culture was initiated from pupae collected in Arusha, Tanzania, in 2002. It was reared for six generations on wheat seedlings at the University of Stirling with no signs of overt NPV infection and then transferred to NERC Centre for Ecology and Hydrology in Oxford. The culture was reared on a semi‐synthetic wheat‐based diet at 28 °C with an 10 : 14‐h light : dark cycle. At each generation, eggs were surface‐sterilized in 1% hypochlorite for 10 min and then 5% formalin for 30 min, in order to remove any potentially contaminating pathogens. Larvae were separated at the second instar and reared individually from this point. Pupae were sexed and mated in groups of around 50 pairs in cylindrical rearing cages and were fed with a 10% honey solution.

### Experimental Design

An experiment was set up to measure three fitness parameters of the virus: mortality and speed of kill, both of which can be used to describe the virulence of the variants, and the number of offspring produced (virus yield) as an estimate of the virus transmission potential. As is standard practice in insect pathology, these metrics were estimated over a range of pathogen doses, because bioassays performed at a single dose can be misleading as they give no indication of the shape or slope of the dose–response curve. The eight variants were compared with the mixed‐genotype, parental WT SpexNPV from which they were derived.

Newly moulted third‐instar *S. exempta* larvae were challenged with five SpexNPV doses: 50, 100, 500, 1000, 5000 OBs per individual. Each dose was given in 1 μL distilled water pipetted onto a small (*c*. 1 mm^3^) plug of artificial diet to individual larvae in square 25‐well plates and left for 24 h. Twenty‐five larvae were infected per dose, except for the lowest two doses where 50 larvae were used in order to produce sufficient numbers of cadavers for the analysis of pathogen yield. An additional 25 larvae dosed with distilled water were used as controls. The virus treatments were replicated twice. All the larvae that had eaten the diet plug in 24 h were transferred into individual 12‐mL pots containing sufficient diet to maintain them through to pupation. Larvae were maintained at 28 °C and were monitored every 8–16 h to produce an estimate of the time taken to kill the larva (infection duration), in addition to virus‐induced mortality. Speed of kill was estimated from the time that the larvae were exposed to the viral dose to when they were observed to be dead. NPV infections are usually obvious as virtually the whole body is converted to virus and the larva becomes pale and flaccid and releases a thick milky fluid when the cuticle eventually ruptures. Any ambivalent deaths were confirmed using Giemsa staining and oil immersion microscopy (×1000). Virus‐killed cadavers were frozen individually at −20 °C in 1·5‐mL Eppendorf tubes until further analysis. Ten cadavers per dose for each virus isolate were selected to estimate the number of progeny OBs produced (virus yield). Only cadavers that could be transferred whole were used for yield analysis. Each dead larva was macerated in 1 mL of sterilized water with a plastic pestle and vortexed thoroughly. OBs from two subsamples were then counted using a 0·1‐mm improved Neubauer haemocytometer and a light microscope at ×400. Four virus‐killed cadavers from each genotype treatment were randomly selected to confirm that the genetic identity of the virus remained constant after the bioassay using RFLP profiling with *EcoR*V (see Redman *et al*. 2010 for methods).

### Data Analysis

Mortality data were analysed using generalized linear models using a binomial error distribution and logit link function. As no control larvae died of virus infection, no correction factors needed to be applied. Non‐viral deaths, predominantly bacterial, did occur at a very low level (<5%) late in the assay, that is after any viral deaths would be expected to occur. Cadavers were stained with Giemsa and checked using a light microscope in case a mixed bacteria–NPV infection was involved; this was not found to be the case, and therefore, these deaths were not included in the virus‐killed total. Speed of kill data (1/number of hours until death) were normally distributed, as was log_10_‐transformed virus yield (number of OBs per larva) and so both were analysed using linear regression. Survival was modelled using a parametric survival model, with an exponential distribution with data censored at 160 h (longer times are likely to be secondary infections). Survival curves were visualized using Kaplan–Meier plots.

## Results

### Comparison of Fitness Traits Among Virus Genotypes

#### Host mortality

Host survival was severely reduced by baculovirus infection. NPV‐induced mortality increased with (log_10_) viral dose (logistic regression: $\chi_{1}^{2}$ = 835, *P* < 0·0001) and differed significantly between the virus genotypes ($\chi_{8}^{2}$ = 65·99, *P* < 0·0001), even after excluding the WT virus from the analysis ($\chi_{7}^{2}$ = 40·58, *P* < 0·0001). The interaction between virus dose and virus genotype was non‐significant in both cases ($\chi_{8}^{2}$ = 7·78, *P* > 0·45, and $\chi_{7}^{2}$ = 3·38, *P* > 0·84, respectively), indicating that all SpexNPV variants responded similarly to viral dose. All SpexNPV genotypes analysed at the end of the bioassay matched those of the virus used for dosing, verifying the stability of the single genotype. As expected, larvae killed by the WT virus treatment produced a profile with multiple submolar bands, indicative of a mixed isolate (data not shown).

**Figure 1:**
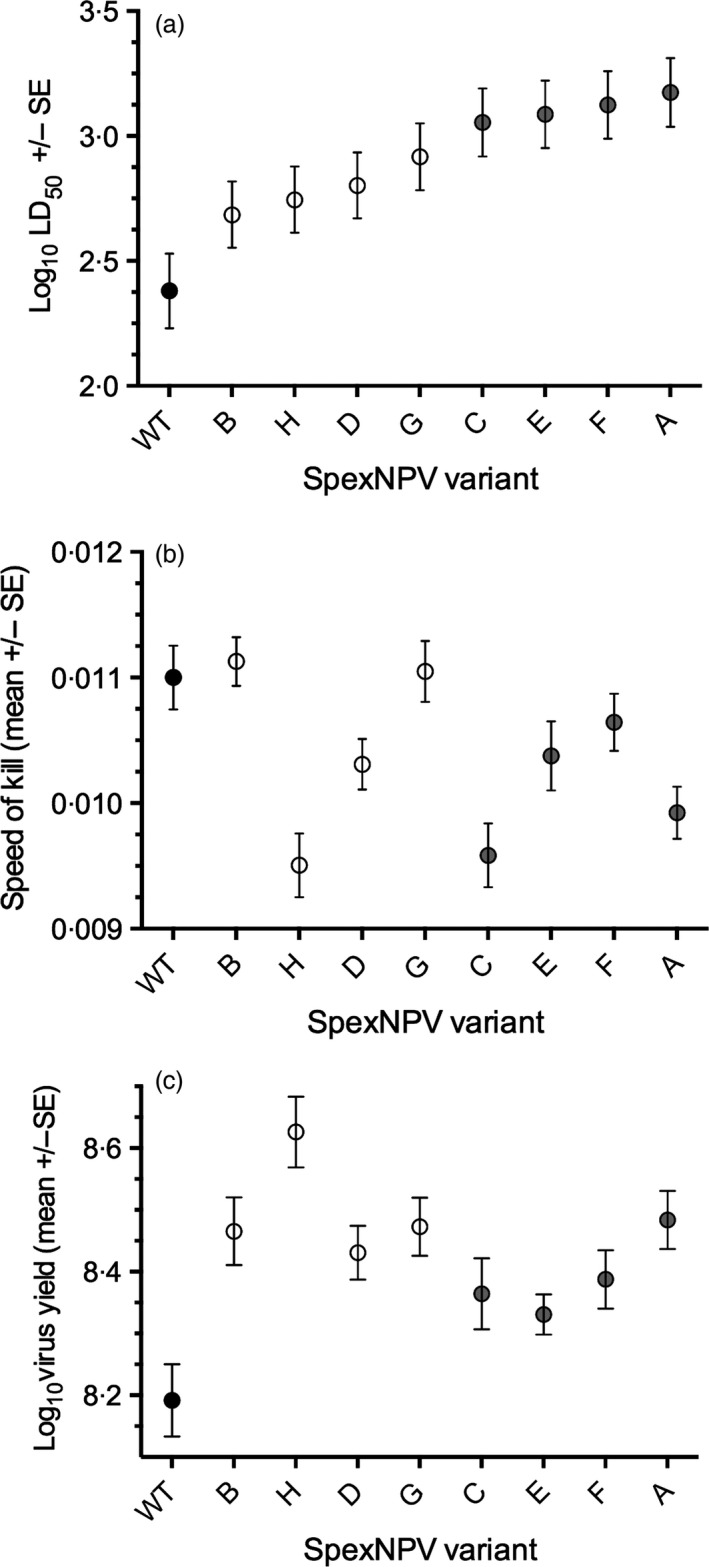
Infection traits for each *Spodoptera exempta* NPV variant (A–H) and the parent wild type (WT). (a) Lethal dose (LD)50s ± standard errors (SEs). (b) Speed of kill (1/infection duration (±SEs), averaged over all virus doses to make it comparable with (a) and (c). Sample size ranged from 209 to 319 individuals per genotype. (c) Mean yield (±SE). Sample size ranged from 19 to 22 individuals per genotype. The black dots represent the wild‐type virus; the white (Group I) and grey (Group II) dots represent the two groups of viruses that can be distinguished in terms of their mortality rate (see text for details).

This variation in virulence of the virus treatments is best illustrated by comparing their LD_50_ (i.e. the estimated number of viral OBs required to kill 50% of inoculated larvae). Across all variants (including the WT virus), there was a sixfold variation, and within the eight SpexNPV single‐genotype variants, there was a threefold difference (Fig. 1a). Model simplification indicated that the single‐genotype variants could be combined into two groups of four without losing any explanatory power: Group I (variants A, C, E and F), with LD_50_s in the range of 1132–1494 OBs per larva, and Group II (variants B, D, G and H), which had consistently lower LD_50_s in the range of 484–825 OBs per larva. The LD_50_ of the parent WT SpexNPV was significantly lower (i.e. more virulent) than any of the individual variants, 240 OBs per larva. After grouping the genotypes in this way, the interaction between virus genotype and dose remained marginally non‐significant (log_10_ dose: $\chi_{1}^{2}$ = 835·64, *P* < 0·0001; variant: $\chi_{2}^{2}$ = 59·64, *P* < 0·0001; log_10_ dose × variant: $\chi_{2}^{2}$ = 4·66, *P* = 0·097; Fig. S1, Supporting information). When the eight single‐genotype variants were combined and compared against the WT parental virus treatment, there was a highly significant difference between the two types of virus treatments, that is multiple‐genotype WT vs. single‐genotype variants (treatment: $\chi_{1}^{2}$ = 24·81, *P* < 0·0001), with the slope of the relationship between virus dose and virus‐induced mortality being steeper for the WT virus (log_10_ dose × variant: $\chi_{1}^{2}$ = 4·66, *P* = 0·025).

#### Speed of kill

Mean time to death across the variants ranged between 89·9 h (variant B) and 105·2 h (variant H); the WT virus was intermediate and took an average of 90·9 h to kill its host. Speed of kill (1/time to death) varied significantly between variants (*F*_8,787_ = 7·24 *P* < 0·0001; Fig. 1b), even after excluding the WT virus (*F*_7,720_ = 7. 39, *P* < 0·0001). However, the speed at which larvae died of NPV infection also increased with increasing viral dose (*F*_1,778_ = 23·58, *P* < 0·0001), and there was a significant interaction between viral dose and virus variant (*F*_8,778_ = 4·03, *P* = 0·0001; Fig. S2), such that the slope of the relationship between viral dose and speed of kill was steepest for the WT virus and shallower for each of the single‐genotype variants (significantly so for variants A, B, C, D and F; as determined by their slope coefficients).

#### Survival

**Figure 2:**
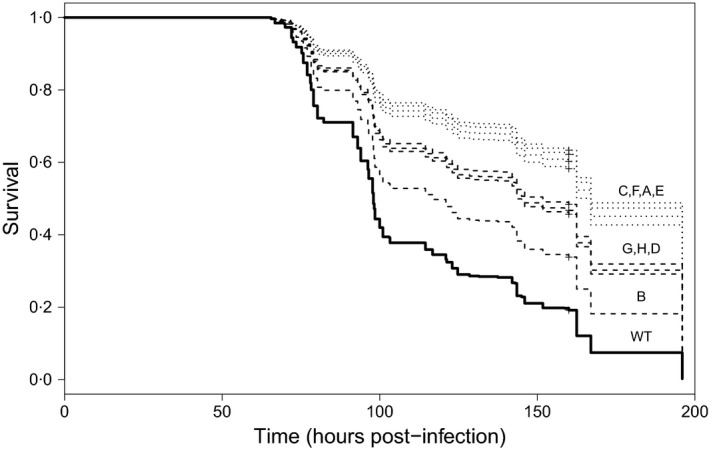
Kaplan–Meier survival curves for individual SpexNPV genotypes (A–H) in comparison with the wild‐type (WT) virus. Each curve shows the fitted values from the survival model standardized for a viral inoculation dose of 1000 OBs. Dotted lines = group I variants (A, C, E and F), and dotted lines = group II variants (B, D, G and H) and the solid line = WT virus.

A survival analysis was conducted to determine the compound effects of virus‐induced mortality and time to death. Overall, there was significant variation between variants in their mortality rates (likelihood ratio test: $\chi_{8}^{2}$ = 50·63, *P* < 0·0001; Fig. 2), with all eight single‐genotype variants dying at a slower rate than the WT virus. Even after excluding the WT virus, there was significant variation between variants in their mortality rates ($\chi_{7}^{2}$ = 30·14, *P* = 0·0002). Mortality rate also increased with increasing viral dose ($\chi_{1}^{2}$ = 675·85, *P* < 0·0001), but all variants responded in a similar manner to viral dose (variant × log_10_ dose: $\chi_{8}^{2}$ = 4·31, *P* = 0·83). Model simplification revealed that the variants could be grouped into the same two clusters, as described for the mortality analysis (see above), with no loss in explanatory power ($\chi_{2}^{2}$ = 46·13, *P* < 0·0001). Thus, larvae infected with the WT virus are predicted to die 2·7 times faster than those infected with variants from Group I (A, C, E, F) and 1·9 times faster than those challenged with Group II variants (B, D, G, H).

#### Production of transmission stages

The number of transmission stages produced varied significantly between variants (*F*_8,170_ = 5·43, *P* < 0·0001; Fig. 1c), even after excluding the WT virus (*F*_7,150_ = 3·56, *P* = 0·0014), which yielded fewer OBs than any of the single genotypes (1·5 × 10^8^ OBs, compared to 2·1 − 4·2 × 10^8^ OBs per larva). Variants E and H differed significantly in the yield produced from the other variants, with E producing significantly fewer and H significantly more (Fig. 1c). Each variant responded similarly to virus dose (variant × log_10_ dose: *F*_8,161_ = 1·29, *P* = 0·25), and there was no change in yield with increasing virus dose (log_10_ dose: *F*_1,161_ = 0·63, *P* = 0·43).

### Virulence–Transmission Trade‐Offs Between Genotypes

#### Within‐variant comparisons

**Figure 3:**
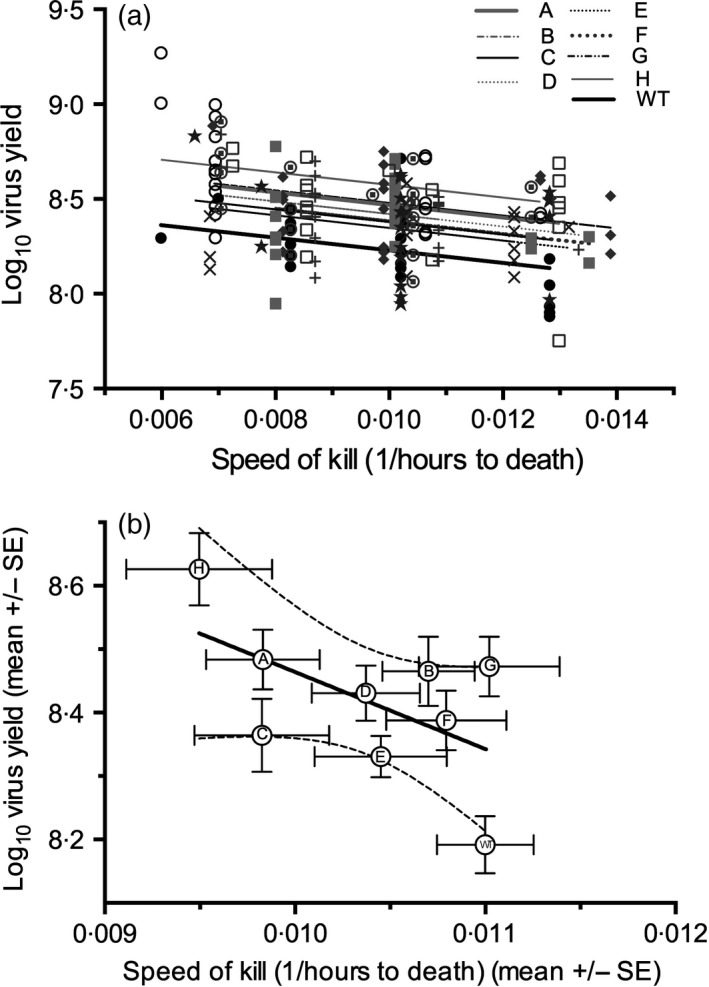
Yield speed of kill trade‐offs for individual SpexNPV genotypes and the wild‐type virus at the (a) intragenotype level (genotype A 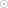, B 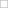, C 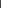, D 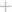, E 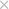, F 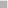, G 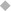, H 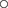 and WT 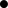) and (b) intergenotype level. In (b), mean speed of kill was estimated only using larvae for which virus yield data were also available.

As predicted, we observed that across all larvae, production of virus OBs (transmission potential) was significantly negatively related to speed of kill (mean slope = −33·34 ± 8·10, *F*_1,169_ = 16·96, *P* < 0·00001; Fig. 3a), consistent with a phenotypic trade‐off between the two traits. Moreover, there was significant variation between virus variants in the elevation of the trade‐off curve (variant: *F*_8,169_ = 4·46, *P* = 0·00006), with the WT virus yielding fewer OBs for a given speed of kill than any of the single genotypes, significantly so for all variants except E (as determined by comparison of the coefficients and their standard errors). The interaction between speed of kill and NPV genotype was non‐significant (*F*_8,161_ = 1·03, *P* = 0·41), as was virus dose (*F*_1,168_ = 2·91, *P* = 0·090). The results remained largely unchanged after excluding the WT parental virus from the analysis (speed of kill: *F*_1,149_ = 11·07, *P* = 0·0011; variant: *F*_7,149_ = 2·36, *P* = 0·025).

#### Among‐variant comparisons

Across genotypes, there was also evidence for a genetic trade‐off between virus yield and speed of kill, but the correlation was marginally non‐significant (Pearson's correlation: *r*_7_ = −0·56, one‐tailed *P* = 0·058; Fig. 3b), although this is largely driven by the fast speed of kill and low yield of the WT virus (after excluding WT: *r*_6_ = −0·23, one‐tailed *P* = 0·29). There was no evidence for a relationship between speed of kill and mortality (LD_50_) (Pearson's correlation including WT: *r*_7_ = −0·40, one‐tailed *P* = 0·14), although the non‐significant trend was in the direction of a faster speed of kill being associated with a lower LD_50_ (i.e. greater mortality). However, an analysis of virus yield vs. LD_50_ indicated a non‐significant positive relationship when the WT was included (*r*_7_ = 24, one‐tailed *P* = 0·74) and a near‐significant negative relationship when it was removed (higher yield associated with greater mortality) (*r*_6_ = −0·56, one‐tailed *P* = 0·076).

## Discussion

The results clearly demonstrate that there is significant genetically based variation between virus variants in natural baculovirus populations in their ability to cause fatal infection, speed of kill and production of transmission stages. At the genotype level, virus dose affects both mortality and speed of kill, with more rapid death at higher doses. This makes biological sense: challenge with low doses of virus is likely to result in the initiation of infection by few virions (Zwart *et al*. 2009), whereas exposure to greater number of OBs will result in multiple foci of infection in the midgut, resulting in a more rapid spread to other tissues. This relationship has been found in other studies looking at more limited numbers of baculovirus variants (e.g. Georgievska *et al*. 2010). However, it was not consistent across all our variants, with some showing no, or even a negative, relationship with speed of kill. This is more curious and might indicate differences in replication speed and tissue tropism, variation in antiviral response at the gut level or interference between virus particles. The lack of a significant relationship between virus dose (mortality) and OB yield suggests that the larvae die when a certain level of OB production is reached (the carrying capacity, see Ebert & Weisser 1997), regardless of how long the infection period is. However, the productivity level reached (virus yield) varies across the different genotypes, perhaps indicating a different rate of tissue conversion or a broader tissue tropism.

We used these data to investigate the interactions among the various components of virus fitness to identify possible trade‐offs and costs to virulence at both the intra‐ and intergenotype level. We measured two aspects of virulence: the level of host mortality induced by the virus and the speed of kill (inverse of the time taken to kill the host). The number of virus occlusion bodies produced was taken as a surrogate for transmission potential, in common with other studies. There was a clear linear relationship between the duration of infection and the production of virus transmission stages for individual genotypes, with OB production steadily increasing as the duration of the infection increased (Fig. 3a). This linear relationship was also close to significance at the intergenotype level (Fig. 3b), suggesting a possible genetic trade‐off. Baculoviruses express a gene that manipulates their hosts (by interfering with larval–larval or larval–pupal moults) to extend their development and body mass, thereby increasing the amount of host tissue that can be converted into virus OBs (O'Reilly & Miller 1989; Wilson *et al*. 2000; Cory *et al*. 2004), suggesting that continued host growth is highly beneficial for virus fitness. In this way, baculoviruses can manipulate the carrying capacity of their hosts. Theory predicts that if there was a cost to prolonged infection, in terms of transmission rate, transmission‐stage production would peak or decelerate with increasing infection duration. There was no evidence for this in the current study. Other studies on single baculovirus isolates or variants also show no indication of costs (Hernández‐Crespo *et al*. 2001; Georgievska *et al*. 2010). However, a nonlinear relationship has been found in another insect–baculovirus system (the pine beauty moth, *Panolis flammea* and *Pf*NPV), in which virus yield plateaued at slower speeds of kill (Hodgson *et al*. 2001). While clearly larval size, and thus OB production, cannot increase indefinitely, there might be differences depending on the stage of the insects, the ecology of the system and the likelihood of transmission (in this case a uni‐voltine forest species *P. flammea* vs. a multi‐voltine agricultural pest *S. exempta*, among other factors) and differing background mortality within the system (Ebert & Weisser 1997).

A key issue here is that baculoviruses are obligate killers, such that horizontal transmission relies on the host dying and releasing occlusion bodies. As a consequence, virulence has to be maximal for baculoviruses; otherwise, horizontal transmission cannot occur, and thus, the assumed trade‐off between exploitation rate and virulence is decoupled. In terms of varying ecologies, one might predict that species with a single generation per year are more dependent on the environmental persistence of OBs to ensure infection the following year, with a larger production of OBs, particularly in later instars, making this more likely. In contrast, multi‐voltine species such as *S. exempta* might benefit from more rapid rounds of infection, although not if the host is continually moving to different areas. This relationship could, however, be modulated by vertical transmission of the virus (Lipsitch, Siller & Nowak 1996). Vertical transmission (from parents to offspring) can occur in baculoviruses, and this appears to be able to take the form of both transmission of an active infection from mother to offspring (most likely through external contamination of the eggs) and via some type of within‐egg transmission which can be either active or quiescent (Kukan 1999; Vilaplana *et al*. 2010; Cory 2015). All types of vertical transmission appear to occur in *S. exempta* (Vilaplana *et al*. 2008, 2010), but their role in virus dynamics in the field and their impact on horizontally transmitted virus has yet to be elucidated in this or any other system.

Trade‐offs between virulence and transmission have been investigated in several other invertebrate obligate pathogen systems. Spore production in one isolate of a bacterial parasite of the waterflea, *D. magna*, showed a humped relationship with host survival time (Jensen *et al*. 2006), implying that intermediate virulence (speed of kill) resulted in higher fitness for the parasite. The lack of a similar intermediate level of virulence in the baculovirus–lepidopteran interaction might relate to the fact that infection and pathogen reproduction only take place in the larval stage, which means that the host can grow considerably and virus replication does not compete with other host processes. *D. magna* intermediate virulence is thought to result from a physiological trade‐off between host and parasite reproduction and a limited growth trajectory for the host. Thus, one might predict that any potential developmental trade‐off in baculoviruses is likely to be age dependent, with the pathogen possibly incurring costs to prolonged host growth only when the larva is approaching pupation. Another factor could be the environments in which these two hosts live. A caterpillar killed with a baculovirus produces a discrete patch of infective OBs with limited opportunities for dispersal. In this situation, bigger is likely to be better as it means more transmission stages. In an aquatic species, such as *D. magna*, patch size and spatial distribution of inoculum might be less important as water will facilitate dispersal. Thus, host ecology and the potentially conflicting demands of within‐ and between‐host dynamics are going to modulate this process.

At the intergenotype level, there were (non‐significant) trends suggesting that higher mortality is positively associated with faster speed of kill and greater yield. However, these relationships, if present, are clearly not strong, and their exploration would require a greater number of virus clones. Few studies have investigated trade‐offs with mortality in obligate killers. In an experimental evolution study, Bérénos, Schmid‐Hempel & Wegner (2009) investigated the co‐evolution of an insect, *T. castaneum* and a fungal (microsporidian) pathogen, *Nosema whitei*, which is an obligate killer requiring host death for spore production and transmission. In contrast to other studies (e.g. Little, Watt & Ebert 2006), pathogen virulence to both the co‐evolved and original insect lines declined during the experiment. One explanation that was proposed was that this reflected a trade‐off between transmission and virulence, as it appeared that high and low levels of mortality resulted in decreased spore loads (Bérénos, Schmid‐Hempel & Wegner 2009). This would imply a trade‐off between the level of host exploitation and the point at which the host is killed (see also de Roode, Yates & Altizer 2008; for another example). Again, this result is likely to depend on the pressures of the normal transmission cycle within the system. The experiment was performed in small vials where transmission is likely to be enhanced. Moreover, in stored products’ insects cannibalism is also often involved in the pathogen transmission cycle. Thus, it is possible that the benefits of maximizing pathogen production for obligate killers are decreased under these circumstances.

One of the most interesting results to emerge from this study is the difference in phenotype between the mixed, WT parental virus and the individual clones that were isolated from it. There was an approximately threefold difference in LD_50_ between the least and the most virulent individual virus variants. However, what was most striking was that the WT virus was significantly more virulent than any of the individual virus variants, with an LD_50_ that was half that of the most virulent variant. This clearly indicates that infections with multiple virus genotypes are much more virulent. All the SpexNPV variants compared in the experiment here were derived from the WT source; however, more than these eight variants are present within the WT mixture (at least 17 distinct genotypes, Redman *et al*. 2010), so there are likely to be other genotypes within the WT mixture that could influence virus phenotype. Recent work on NPVs isolated from other *Spodoptera* species has demonstrated that natural baculovirus populations can also contain defective genotypes which lack certain virulence genes, making them incapable of infection on their own. Mixed infections with these defective viruses have shown that they can both increase (López‐Ferber *et al*. 2003) and decrease (Muñoz & Caballero 2000) the resulting level of host mortality and that these effects are dependent on the combination of variants (Simón *et al*. 2005).

Mixed‐genotype infections and their impact on virulence have recently received a lot of attention (e.g. Alizon, de Roode & Michalakis 2013; Ben‐Ami & Routtu 2013). One hypothesis relating to mixed infections is that faster‐growing strains are favoured and increased competition could lead to increased host mortality. While mortality is clearly increased in the mixed WT treatment, this does not appear to alter speed of kill. The speed of kill of the WT isolate was rapid but on a par with several other single variants; however, rather surprisingly, the virus yield was significantly lower than that of any other variant. In fact, one variant, ‘H’, produced a yield that was 2·8 times greater than that of the wild type. While the relationship between speed of kill and virus productivity (rapid death – low yield) is relatively (but not totally) consistent at the level of the individual genotype, WT virus mixtures do not always conform to this. Field‐collected isolates of NPVs from both the western tent caterpillar *(M. californicum pluviale)* and the winter moth (*O. brumata*) show that WT isolates can be both fast to kill and retain high productivity (Raymond *et al*. 2002; Cory & Myers 2004). Why the SpexNPV WT is different is unclear. One explanation for the decreased production of transmission stages could be that there is interference or exploitation competition between the variants, resulting in an overall reduction in virus replication and OB production. In particular, it is possible that any defective genotypes present in the wild type could have a detrimental effect. If this is the case, it might be expected that yield would decrease with increasing dose, as a higher dose is likely to result in more initial foci of infection. However, this does not occur, which might indicate that the interactions take place later in the infection process. Alternatively, the different yield speed of kill relationships could result from the varying demands of species with very different life cycles and phenologies (uni‐voltine temperate forest insects vs. multi‐voltine tropical species). Understanding the impact of mixed infections on virulence in obligate‐killing baculoviruses is likely to require studies which mimic more realistic ecological scenarios. In particular, successful transmission to a new host is the result of more than just producing many transmission stages, and lifetime fitness of a parasite needs to take into account the between‐host component. Only further field‐based experiments with realistic estimates of transmission and environmental persistence will tease apart these interactions and identify the factors which determine the structure of pathogen populations and their impact on the evolution of virulence.

## Data accessibility

Data available from the Dryad Digital Repository: http://dx.doi.org/10.5061/dryad.427m6 (Redman, Wilson & Cory 2016).

## Supporting information


**Fig. S1.** Effect of the interaction between viral group and viral dose on virus‐induced mortality.
**Fig. S2.** Effect of the interaction between virus variant and viral dose on speed of kill.
