# Supplementary material for: Trade‐offs and mixed infections in an obligate‐killing insect pathogen
Source: J Anim Ecol. 2016 Jun 13;85(5):1200–9. doi: 10.1111/1365-2656.12547 (PMC4988505; doi:10.1111/1365-2656.12547)

**Supplementary figures**

Figure S1. Effect of the interaction between viral group and viral dose on virus-induced mortality. The points are the logit-transformed mean values of the raw data (black = wild-type virus, grey = group II variants B, D, G and H, white = group I variants A, C, E and F). The lines are the fitted values from logistic regression model (solid = wild-type, dashed = group II variants, dotted = group I variants). The interaction was marginally non-significant when the three groups were compared (p=0.097), but marginally significant when the wild-type virus was compared to the variants (groups I and II) combined (p = 0.025; see main text). Mortality for the wild-type virus at the highest dose was 100% and hence logit mortality was equal to infinity, but for illustrative purposes it is shown here as equalling 4 (equivalent of >98% mortality)


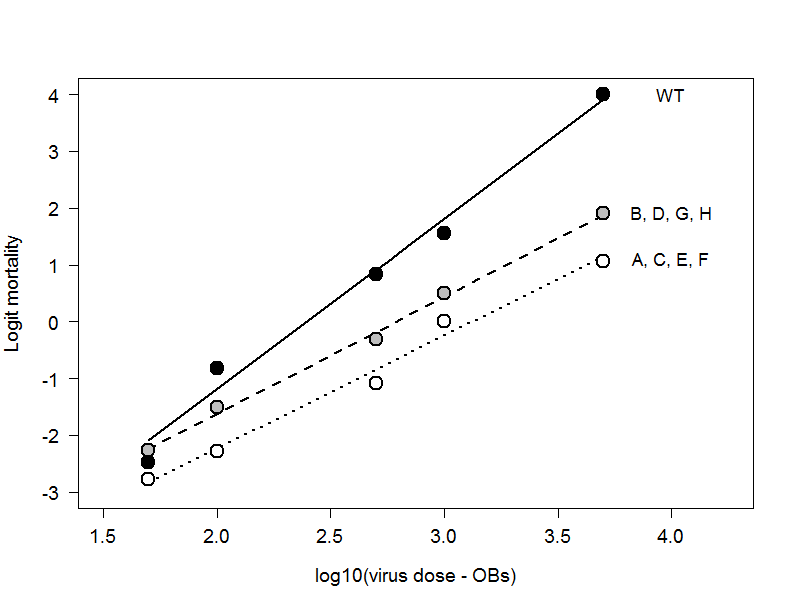


Figure S2. Effect of the interaction between virus variant and viral dose on speed of kill. The points are the mean values of the raw data. The lines are the fitted values from linear regression model. The variant*dose interaction term was statistically significant (p = 0.0001; see main text).


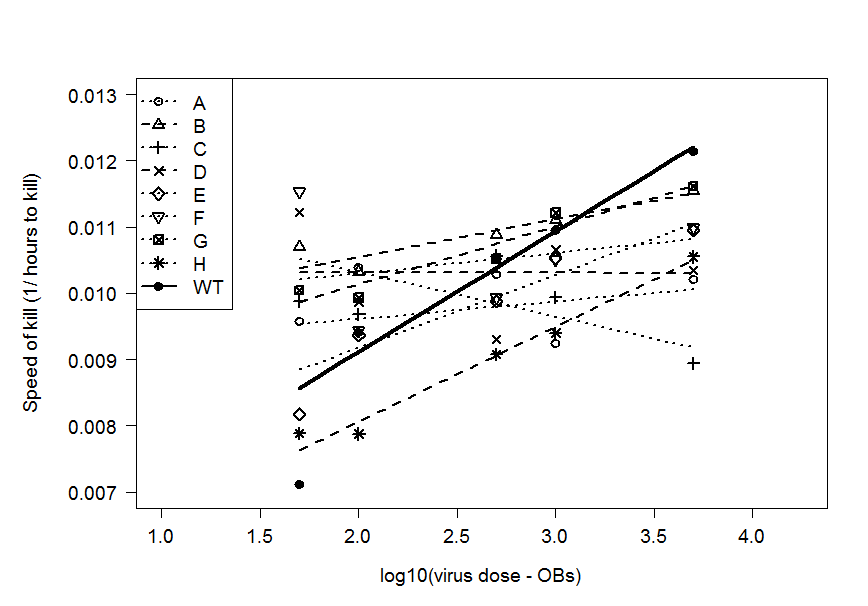

Supplement: Supplementary file 1 — Fig. S1. Effect of the interaction between viral group and viral dose on virus‐induced mortality. Fig. S2. Effect of the interaction between virus variant and viral dose on speed of kill. [file JANE-85-1200-s001.docx]
